# Differential effect of motivational features on training improvements in school-based cognitive training

**DOI:** 10.3389/fnhum.2014.00242

**Published:** 2014-04-24

**Authors:** Benjamin Katz, Susanne Jaeggi, Martin Buschkuehl, Alyse Stegman, Priti Shah

**Affiliations:** ^1^Combined Program in Education and Psychology, Department of Psychology, University of MichiganAnn Arbor, MI, USA; ^2^School of Education, University of California at IrvineIrvine, CA, USA; ^3^MIND Research InstituteIrvine, CA, USA; ^4^Department of Psychology, University of MichiganAnn Arbor, MI, USA

**Keywords:** working memory, intervention, motivation, video games, n-back

## Abstract

Cognitive training often utilizes game-like motivational features to keep participants engaged. It is unclear how these elements, such as feedback, reward, and theming impact player performance during training. Recent research suggests that motivation and engagement are closely related to improvements following cognitive training. We hypothesized that training paradigms featuring game-like motivational elements would be more effective than a version with no motivational elements. Five distinct motivational features were chosen for examination: a real-time scoring system, theme changes, prizes, end-of-session certificates, and scaffolding to explain the lives and leveling system included in the game. One version of the game was created with all these motivational elements included, and one was created with all of them removed. Other versions removed a single element at a time. Seven versions of a game-like n-back working memory task were then created and administered to 128 students in second through eight grade at school-based summer camps in southeastern Michigan. The inclusion of real-time scoring during play, a popular motivational component in both entertainment games and cognitive training, was found to negatively impact training improvements over the three day period. Surprisingly, scaffolding to explain lives and levels also negatively impacted training gains. The other game adjustments did not significantly impact training improvement compared to the original version of the game with all features included. These findings are preliminary and are limited by both the small sample size and the brevity of the intervention. Nonetheless, these findings suggest that certain motivational elements may distract from the core cognitive training task, reducing task improvement, especially at the initial stage of learning.

## INTRODUCTION

A key challenge in cognitive training research is how to keep participants engaged in training. Training programs are often challenging for participants to complete, and it is expected that they will remain focused on a task or set of tasks for 20–40 min at a time (Jaeggi et al., 2008; Thompson et al., 2013), for anywhere between a few days (Rueda et al., 2005) to 100 sessions (Schmiedek et al., 2010). Because transfer improvements generally require several hours of training (Jaeggi et al., 2008; Stepankova et al., 2013) it is important that participants in training paradigms remain compliant during training. Additionally, it may be necessary for participants to improve in the training program in order to experience transfer on untrained tests (Jaeggi et al., 2011).

Unfortunately the time commitment and effort required to complete a cognitive training study is often such that many participants do not complete the experiment. Studies often have high dropout rates, including some higher than 25% (Redick et al., 2013; Jaeggi et al., 2014). A variety of individual difference factors may contribute to a participant’s ability to successfully engage in and complete the training, such as baseline ability in the training task and one’s intrinsic motivation to complete a training program (Jaeggi et al., 2014).

While individual difference factors are generally outside of the experimenters’ control, the design of the training program may also contribute to a participant’s engagement in the task, and these game design elements are often relatively simple to adjust. Cognitive training paradigms vary widely in the type of motivational elements they include, however, and while some studies have focused on recruiting unpaid, intrinsically motivated individuals that may be more likely to engage with and complete a training regimen (Jaeggi et al., 2014), others have utilized substantial financial compensation as a means of encouraging participants to complete the training (Redick et al., 2013; Thompson et al., 2013). Factors that may impact a participant’s ability and willingness to engage, comply, and improve in training have been the subject of some interest in recent research. Studies with children often utilize prizes, certificates, and display of high scores to encourage individuals to excel at and complete the training (Holmes et al., 2009; Jaeggi et al., 2011; Wang et al., 2014).

One topic that has not gotten much attention is how game-based motivational elements may contribute to improvements in training and transfer. This is somewhat surprising, considering that elements such as score, tutorials and scaffolding, theming, and feedback are often prominently featured in cognitive training programs. Cognitive psychologists and neuroscientists often find themselves in the role of game designer (Mané and Donchin, 1989; Anguera et al., 2013), and even some of the most basic training paradigms have at least included a motivational chart showing player improvements (Jaeggi et al., 2008). Other training programs, particularly those targeted at children (Klingberg et al., 2005; Jaeggi et al., 2011) look and feel more like traditional video games with appealing art and sound design. Cognitive training games are similar to certain types of entertainment games – specifically, those that Gee (2006) would describe as “problem games,” – that involve simple, repetitive mechanics, rather than large, open worlds for the player to explore. Almost all tasks used in cognitive training games, from n-back to useful field of view to conflict resolution tasks, can be translated into fairly simple gameplay mechanics (Klingberg et al., 2005; Rueda et al., 2005; Ball et al., 2010; Jaeggi et al., 2011; Alloway, 2012).

While game-based motivational elements have not been well-studied within cognitive training research, some of them have been examined by learning game researchers. For example, one popular game element, persistent scoring (the presentation of a number that represents player performance and changes as the player completes the task successfully) likely encourages engagement and motivation (Toups et al., 2009). However, the way this scoring is implemented, that is, whether points are earned specifically for completing tasks essential to the learning goal or are awarded for other non-core actions, can determine if scoring hinders or helps learning on the task (Habgood and Ainsworth, 2011). The inclusion of game features may either support or subvert participant motivation to engage depending on how well they tie in with the learning task and the participant’s pre-existing motivational framework. For example, imagine a cognitive training game that includes an extra bonus round where players perform some other task non-essential to the training component, such as answering a trivia question. If the number of points possible for the bonus round matches or is greater than that awarded during the core task, participants may be less motivated to perform well during the training portion of the game. This contrasts with situations where the reward is directly reinforcing of the performance task. In one related example, a review of reading incentive programs supported using literacy-related reward to motivate students (Fawson and Moore, 1999); one study found that students who received a book as a reward following a reading program were more motivated to participate than those who received a token prize (Marinak and Gambrell, 2008).

Psychologists who study motivation are also interested in game-based motivational features (Ryan et al., 2006; Przybylski et al., 2010), possibly because games are an ideal context for understanding the tension between intrinsic motivation and extrinsic reward. Elements such as scoring and feedback may impact a player’s intrinsic motivation and may also contribute to their success in learning the content included in the game. For example, in Malone’s examination of intrinsically and extrinsically motivating game elements, different versions of the game *Breakout* were created that included elements of feedback such as persistent score and breaking bricks (Malone, 1981). Versions of the game with both of these elements were rated much more highly on a scale of enjoyment by players than versions where they were not present. Theming (referred to as “fantasy” by Malone) was also evaluated and found to significantly contribute to a child’s interest in the game, although gender differences were identified in the type of theme each child enjoyed the most.

More recently some focus has been applied to issues of motivational game elements in cognitive research, however, the research has thus far been inconclusive. Two recent studies compared game versions that included a variety of motivational elements such as those studied by Malone, to more basic versions of a task. While Prins et al. (2011) found that including game elements such as theming, game-like feedback, and animations increased motivation as well as performance for children completing a working memory training game, recent work from Hawkins et al. (2013), found that the addition of similar game features improved the player experience but not the quality of data collected during a cognitive task. One possible explanation of these mixed results is that the amount of time spent with the game experience also matters – while Prins et al. (2011) examined the effect of game features over 3 weekly sessions, the Hawkins et al. (2013) study included one single session of play for the games used.

It is also possible that the impact of scoring and other game-like features may differ from game to game, depending on factors such as the goals, difficulty, and demographics of the users. Conclusions drawn from one study cannot necessarily be applied more generally to other types of games or interactive experiences. Nevertheless, no study thus far has systematically examined the impact that *individual* game elements, rather than several features together, have on player performance; previous studies such as those from Hawkins et al. (2013) and Prins et al. (2011) compare versions of the game with a variety of features to versions of the game without any features present. Therefore findings from this present research will be of considerable interest to game designers beyond the cognitive training space. By separating out the most popular game-elements included in these training games, such as scoring, lives and leveling, prizes, and theming, we may better understand the extent that these elements contribute to participant engagement and improvements on the task.

To examine how these elements impacted performance on a visuospatial working memory training task, we designed several versions of a three-day working memory game based on a cognitive training task used in previous research (Jaeggi et al., 2011). In the original version of the task, many motivational elements were included, such as changing themes and art, display of score, lives and levels, and prizes and certificates awarded for player compliance and performance. We created new versions of this game, each with one of these elements removed, as well as one with several game-like motivational features absent from the training task. Even without persistent score, lives, prizes, and changing theme, the task was still *game-like*, with whimsical art and scoring presented between rounds.

This point brings up a significant additional note: why versions of the game with a single element removed were created rather than several versions with one single element added to a bare-bones version of the task. This would likely have been the approach taken if the experimenters had created a completely new game, however, each version of the task is a modification of a training game used in a previous cognitive training study (Jaeggi et al., 2011). Removing a single element generally did not impede gameplay but some elements are interdependent with each other. For example, the prizes students could pick at the end of each day in most conditions were offered based on the total score; students with a higher score could pick prizes of greater value. While other types of feedback (such as the display of leveling on screen) still gave sense of their performance and could be connected to earning better prizes, the addition of performance-based prizes without *any* additional context may not have made sense to the player. In this study the question of interest was whether removing any additional feature might have significant effect on motivation or training gain, and thus each version had one element removed. However, an alternative design, where a single feature is added to a completely bare-bones version of a task, offers an interesting possibility for future research.

We hypothesized that there would be a differential effect of motivational feature for learning on the training task. For example, given existing research on the potential negative effects of extrinsic reward, such as Marinak and Gambrell (2008), we expected that the removal of prizes might increase learning on the training task. However, in general, the findings from the Prins et al. (2011) study led us to expect that students in the group with all motivational elements included would outperform students in the no motivational element group. Additionally, students in a previous study using the same version of the game as in the “all motivational features” group who reported greater enjoyment of the task outperformed those who did not enjoy the task as much (Jaeggi et al., 2011). The results from Hawkins et al. (2013) and Prins et al. (2011) suggested that students in the group with the most motivational elements would rate more highly on self-report measures of intrinsic motivation or enjoyment; it is possible that the versions of the game that students enjoyed more would also be the versions where they experienced greater improvement on the training task. Thus we expected that removing other features commonly included in games, such as changing theme, scoring, and lives and levels would have a deleterious effect on learning the training task.

We included an outcome measure relatively similar (but not identical) to the training task, in which players were required to identify if a given object presented on screen matched an object presented on screen *n*-items earlier. Despite the similarity between the transfer task and the outcome measure, we did not expect to see significant transfer gain due to the limited three-day training duration. Rather, we primarily expected to find differences in player self-reported and observed motivation and performance on the task based on which elements were excluded. We hope that a better understanding of how the game-like elements included in this study impact motivation and performance will help researchers design better, more scientifically useful, cognitive training paradigms.

## MATERIALS AND METHODS

### PARTICIPANTS

128 students were recruited from seven different school-based summer camps in the southern Michigan region (average age = 10.56 years, SD = 2.48, range 5–14, 37% girls). Students were invited to participate in a three day long experiment in which no compensation was provided outside of the possibility of prizes or certificates in some variants of the intervention; recruitment occurred at tables outside the entrance to the summer camps immediately prior to the start of each camp. Written informed consent was collected from both parents and students prior to participation. Students were also asked if they wished to continue the experiment prior to each training session and were informed that they could end their participation at any time. 21 students were not included in the analysis due to either not completing the entire three days of training and testing (*N* = 13), having taken part in previous cognitive training research (*N* = 2), or being too young to be included in the study (younger than 6 years, *N* = 6). Of the 13 students who dropped out and were not too young or participants in previous cognitive training research, no more than four dropped out of any individual condition. 107 students (average age = 10.65 years, SD = 2.36, range 6–14, 44% girls) were then included in the final analysis. Because students completed the tests, questionnaires, and training together as part of the camp, game versions were assigned randomly at the camp level to avoid children comparing the game and prizes amongst themselves and perhaps being disappointed when some received prizes or played more engaging games than others. Running the experiment within summer camps enabled us to evaluate motivational features in a real-world environment, however, one trade-off of this approach is that group sizes and ages differed somewhat depending on which camp students were recruited from. The demographic information for each condition is included in **Table 1**.

**Table 1:** Demographic information.

Condition	*N*	Age (years)	Grade
All optional features included	25	11.28, SD 2.82	6.00, SD 2.38
No theme change	13	10.92, SD 1.71	5.77, SD 1.54
No points shown	19	12.21, SD 2.20	6.84, SD 1.64
No prizes	15	8.40, SD 2.03	3.33, SD 1.63
No explanation of lives/levels	11	9.82, SD 1.72	5.00, SD 1.61
No explanation of lives/levels or certificates	12	10.83, SD.835	5.42, SD 0.52
No optional features included	12	10.00, SD 1.86	4.83, SD 1.64

### PROTOCOL

**FIGURE 1:**
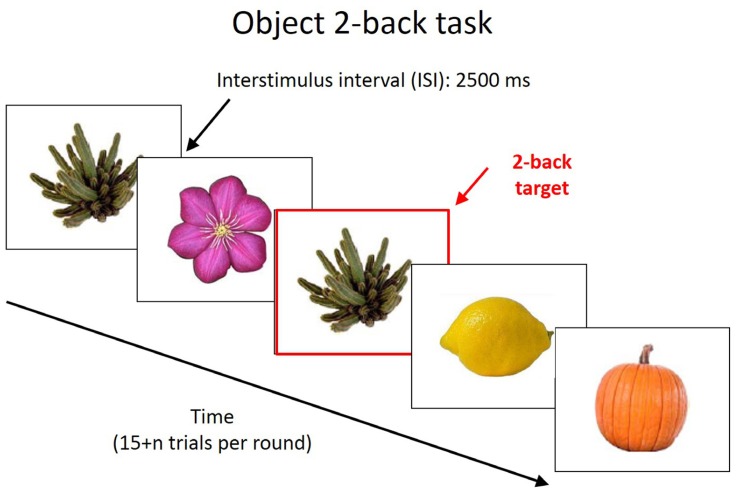
**Object 2-back task.** Participants were presented with a sequence of pictures and they were instructed to indicate whether the current picture was the same as the one 2 pictures back in the sequence.

A pre-test was administered on the first day of the experiment prior to the training (**Figure 1**). The pre-test consisted of a computerized object 2-back assessment that presented participants with a sequence of images one at a time. Participants were required to determine whether each item matched the one presented two items previously and then press one of two keys to indicate their answer. An object was presented every 3 s, with a presentation time of 500 ms and an inter-stimulus interval of 2,500 ms. The pre-test consisted of three blocks of 17 stimuli each and performance was measured as the proportion of correct answers minus the proportion of false responses. Each block included five target trials and 10 non-target trials after the presentation of the initial two stimuli. A few practice trials were included prior to the actual assessment to ensure that the children understood how to complete the task. Following the pre-test on the first day of the study, students began training with the n-back working memory game. After the training on each day, experimenters orally administered brief surveys with Likert-type questions asking how much students enjoyed the game, how exciting the game was, how difficult the game was, and how much effort each student had put into the game. These four questions were adapted for a previous cognitive training study from a factor analysis of the Intrinsic Motivation Inventory (McAuley et al., 1989; Jaeggi et al., 2011). Each of these variables was averaged over the course of three-days to create enjoyment, excitement, effort, and difficulty variables. Researchers also rated students on how engaged they seemed during each day of training using a Likert-type scale following each training session; this was also averaged over the course of the three days to create a final observer engagement score for each participant. Following the third day of training, participants completed the object 2-back assessment a second time.

#### Cognitive training game

Participants trained on a game-like computerized working memory task similar to that used in a previous study with children (Jaeggi et al., 2011). This spatial n-back task presented participants with stimuli at one of six locations on the screen, at a rate of 3 s each, with 2,500 ms between stimuli and with each stimulus presented for 500 ms. Students were required to press the A key each time the current stimulus matched the location of the one presented *n* items previously, and the L key each time the current stimulus did not match. Participants completed 10 rounds of this task each day, each round consisting of 15 + *n* trials, and each round consisting of five targets and 10 + *n* non-targets. All versions of the game were adaptive in that the *n* level was adjusted depending on performance in each round. If a participant made four or more errors they would lose a single life; after losing three “lives” the participant’s *n*-level would be decreased by 1 in the following round. If a participant made three or less errors *n* increased by 1 in the following round.

Seven versions of the n-back training game was developed to examine the role of five motivational features: points, theming, explanation of lives and levels, prizes, and end-of-session certificates. One version of the game included all of these motivation features, while another included none of them. Four of the other versions excluded one of these features. Due to experimenter error, one additional version that was meant to exclude the certificates provided to players at the end of each session also excluded the display of lives and levels feature. However, because this group (with two interrelated elements) was of potential interest, it was included in the subsequent analysis.

**FIGURE 2:**
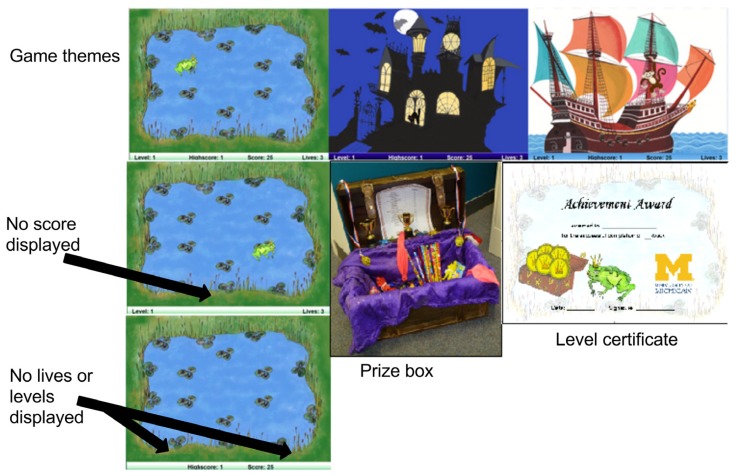
**Game elements.** Shown here are the three versions of the game theme, the appearance of the game in the original theme if points or lives and levels were removed, the prize box and a sampling of prizes given, and the level certificate template used in the study.

***Theming.*** Several different themes were developed to make the n-back task more appealing to students, that is, a frog jumping on lily pads, a cat appearing in windows of a haunted house, and a monkey jumping from sail to sail on a pirate ship (**Figure 2**). In all game versions except for the one excluding theming, the theme changed before the first round on the second and third day of training. In the “no theme” group as well as the “no motivational features” group, only the lily pad theme was included, and this theme remained persistent across the three days of training.

***Score.*** A bar on the bottom of the screen displayed score as the player completed the n-back task. Points were earned for correctly identifying whether the location of the character on the screen matched the location presented *n* instances earlier. In versions of the game with prizes, players were instructed that they could trade in points earned for a prize at the end of each day. In the “no points” and “no motivational features” versions of the game, the persistent score was hidden during play (**Figure 2**). The score was still shown at the end of each round, however.

***No display or explanation of lives or levels.*** Lives left and the current level were displayed on-screen during play. “Levels” indicated the *n* level the user was currently on, while “Lives” was used to indicate how many errors the participant could make before dropping an n-back level on the subsequent round. The “Lives” and “Levels” indicator were hidden on the bottom bar (**Figure 2**) for the “no lives or levels explanation” group, as well as the “no motivational features” group. Additionally, in these groups the experimenter did not mention lives and levels. The game remained adaptive as in the other conditions, however, and the participants still received a certificate after each day’s training with the n-back level he or she had reached.

***Prizes.*** Prizes were offered each day after the completion of the game in exchange for “points” the participants had earned. In the “no prizes” group and the “no motivational features” group, participants were given a prize at the very end of the study, but not each day during training. Additionally, participants in those groups were not told that prizes would be given prior to completing the post-test on day three. In the groups where prizes were present, students were allowed to see a treasure box (**Figure 2**) from which they would select items at the end of each day.

***End of session certificates and no display or explanation of lives and levels.*** Players were awarded a certificate (**Figure 2**) at the end of each training day celebrating the level they reached. In the “no certificate” version of the game players were supposed to complete the standard version of the task but without a certificate given at the end of the round, however, the experimenters for this group incorrectly administered a version of the game without the display of lives or levels. Thus players in one of the seven groups were not aware of the role of lives or levels during the task, and additionally did not receive certificates at the end of each day

## RESULTS

To identify differences in motivation, training performance over time, and pre/post-test performance on the object n-back measure, omnibus analysis of covariances (ANCOVAs) were conducted with all game conditions included; in the case of a significant effect of game-type on these variables, follow-up ANCOVAs were conducted comparing each game variant to the original version with all features included. Despite attempts to recruit summer camps with similar ages, there were significant differences in age across some of the training groups *F*(6,100) = 5.46, *p* < 0.001, partial η^2^ = 0.247. Age predicted improvement in the training following a regression analysis including the age of the pooled participants as predictor and the rate of improvement (operationalized as the slope of a linear model – see also below) of the task as outcome, β** = –0.202, *t*(105) = –2.108, *p* < 0.05, *R*^2^ = 0.041 (proportion of variance in slope explained *F*(1,105) = 4.444, *p* = 0.037). Thus, we included age as a covariate in our subsequent analysis.

### TRAINING PERFORMANCE

**FIGURE 3:**
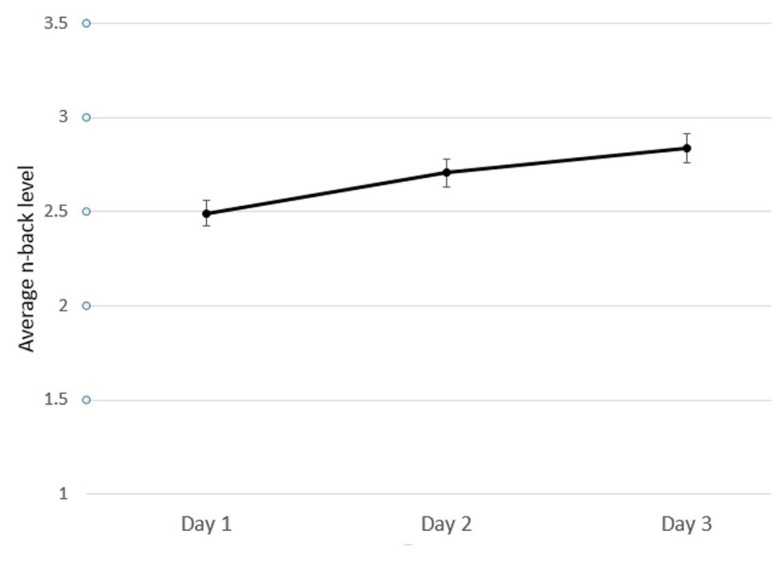
**Average game performance (n-back level) across all individuals on each day.** Error bars represent standard error.

**FIGURE 4:**
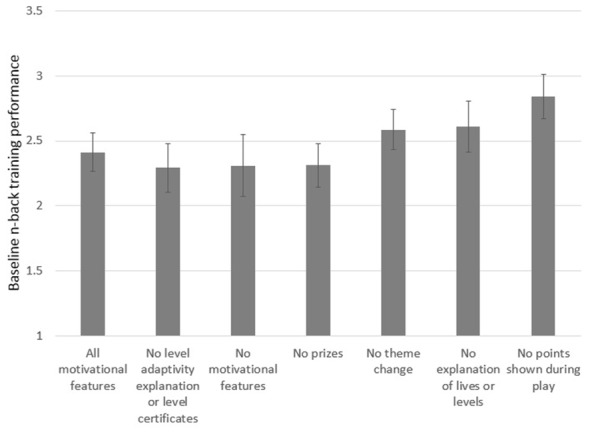
**Average game performance (n-back level) at baseline for each game version.** Error bars represent standard error.

To quantify each participant’s training improvement over the three sessions of training, the slope of a linear regression model was calculated for each participant using the average n-back level per day of training (**Figure 3**). Due to the difference in ages across conditions (**Table 1**) and the variance in baseline performance across game versions (**Figure 4**), we included age and starting n-back level as covariates in our analyses. A univariate ANCOVA across conditions revealed a significant effect of game-version on training improvement as measured by linear slope *F*(6,98) = 2.49, *p* = 0.028, partial η^2^ = 0.132).

**FIGURE 5:**
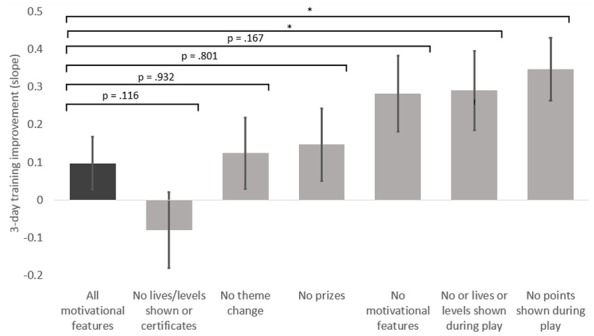
**Adjusted means of training slope by game-type controlling for age and baseline n-back level.** Means are estimated at age = 10.65 and baseline n-back = 2.49. Error bars represent standard error. *p < 0.05.

To analyze the effect of each individual motivational feature on performance, we then computed a set of univariate ANCOVAs with training slope as the dependent variable, calculated from the average n-back on each day of the training task. We compared students playing the version of the game with the full set of motivational features to students playing each of the other versions with elements removed; see **Table 2** and **Figure 5**. Students who played the version of the game without the persistent display of score performed significantly better at the training task over time versus students who played the version of the game with all motivational features, *F*(1,40) = 7.22, *p* = 0.010, partial η^2^ = 0.153) as did students who completed the version of the game without the indication of lives or levels, *F*(1,32) = 4.48, *p* = 0.042, partial η^2^ = 0.123. However, students in the group without theme changes did not perform significantly different from the group with all motivational features *F*(1,34) = 0.07, *p* = 0.801, partial η^2^ = 0.002), nor did the group that did not receive prizes after each training session *F*(1,36) = 0.01, *p* = 0.932, partial η^2^ = 0.000). The group that did not receive certificates after each day, and also did not see lives or level information during gameplay, trended worse than the all motivational features group, but not significantly so, *F*(1,33) = 2.60, *p* = 0.116, partial η^2^** = 0.073. The group that completed the version with no motivational features trended higher but did not differ significantly in performance on the training task from the group with all features, *F*(1,33) = 2.00, *p* = 0.167, partial η^2^ = 0.057).

**Table 2:** Training improvement by game variant.

Condition	*N*	Estimated mean slope	Standard error	*p*	partial η^2^
All motivational features	25	0.10	0.07	–	–
No certificates given and no lives or levels displayed	12	-0.08	0.10	n.s.	0.07
No theme changes	13	0.12	0.10	n.s.	0.00
No prizes awarded	15	0.15	0.10	n.s.	0.00
No points displayed	19	0.35	0.08	*	0.15
No lives or levels displayed	11	0.29	0.10	*	0.12
No motivational features	12	0.28	0.10	n.s.	0.06

Slope here represents the linear model of training improvement calculated using the average n-back performance during each daily session. Slope is controlled for age and baseline n-back level and means are estimated at age = 10.65 and baseline n-back = 2.49. Significance and effect size are drawn from each follow-up ANCOVA that compares a single condition to the original, all motivational features group. Only the “No points displayed” and “No lives or levels displayed” differed significantly from the “all motivational features” comparison group. *p < 0.05.

An additional analysis was performed to further examine the most robust finding, that the display of points on screen may have had a deleterious effect on game performance, as well as to partially address the issue of small samples sizes in the study. A final univariate ANCOVA was thus conducted in a similar manner as above with both the group without any motivational features and the group with only the score removed (*N* = 31) compared to all other participants (*N* = 75, all of whom played a version of the game where points were displayed). This analysis further supported the original finding, as the combined task performance of all individuals who did not see points displayed was significantly better than the combined performance of all individuals who did have points displayed *F*(1,103) = 7.937, *p* = 0.006, partial η^2^ = 0.072.

### MOTIVATION

Participant self-ratings of task-related enjoyment, difficulty, effort, and excitement were averaged over the three days and examined as a function of game variant. ANCOVAs with game-type as the independent variable and age as a covariate did not find a significant effect of game-type on student self-report of enjoyment, *F*(6,98) = 1.52, *p* = 0.180, partial η^2^ = 0.084, excitement, *F*(6,98) = 1.43, *p* = 0.188, partial η^2^ = 0.080, or effort, *F*(6,98) = 1.35, *p* = 0.241, partial η^2^** = 0.076, or student self-report of difficulty, *F*(6,98) = 1.94, *p* = 0.082, partial η^2^ = 0.105. However, as in other studies of motivational factors in differently aged students (Lepper et al., 2005), a median split of students by age revealed that students 10 and under (*N* = 47, mean = 3.75, SD = 0.79) were significantly more excited to complete the task than students 11 and older (*N* = 60, mean = 3.29, SD = 0.72) to complete the task [*F*(1,103) = 9.78, *p* = 0.002, partial η^2^ = 0.085]. On self-ratings of enjoyment, younger students (*N* = 47, mean = 3.89, SD = 0.54) were also more likely than older students (*N* = 60, mean = 3.57, SD = 0.67) to enjoy the task, *F*(1,103) = 7.38, *p* = 0.008, partial η^2^ = 0.066, suggesting at least that the student questionnaires did accurately capture their personal feelings regarding engagement with the game. Additional analyses of motivational factors for the combined game versions without the display of points compared to the game versions with points on screen did not identify a significant impact of this feature on any of the motivational factors, although students in the group that did not see a persistent score reported applying marginally less effort during gameplay (*N* = 31, *M* = 3.76, SD = 0.55) than those who did see a score (*N* = 75, *M* = 3.48, SD = 0.67), *F*(1,103) = 3.901, *p* = 0.051, partial η^2^ = 0.036. Averaged observer ratings of player engagement over the three-days were also examined as a function of game variant. Again, an ANCOVA was conducted including researcher engagement ratings as the dependent variable, game-type as the independent variable, and age as a covariate. Game-type did not significantly predict experimenter ratings of engagement, *F*(6,99) = 1.91, *p* = 0.086, partial η^2^ = 0.104.

### OBJECT n-BACK TRANSFER TASK

Finally, performance on the object 2-back near-transfer task was examined through an ANCOVA with gain on the object n-back test as the dependent variable, game type as the independent variable, and age and pre-test performance on the object 2-back task as covariates. No differences in improvement were identified between any of the game variants, *F*(6,98) = 1.54, *p* = 0.175, partial η^2^ = 0.086. There was a marginal effect of having score displayed when all individuals who played a version without persistent scoring (*N* = 31, mean object n-back gain = 0.06, SD = 0.21) were compared to the combined participants training with a version with persistent score (*N* = 75, mean object n-back gain = 0.02, SD = 0.29), *F*(1,103) = 3.070, *p* = 0.083, η^2^ = 0.029. This is not surprising, however, as the training regimen were likely too short for sizable near-transfer effects to occur. The untrained object n-back task performance across all participants was not significantly higher after only three days of training (*M* = 0.473, SD = 0.258) than at the start (*M* = 0.443, SD = 0.221), as revealed through a paired-samples *t*-test, *t*(106*)* = 1.14, *p* = 0.255.

## DISCUSSION

The results of this research should add nuance to our understanding of how popular “motivational” game features impact actual player performance. Over the three days of the study, students playing versions of the game without the persistent display of points and without the display of lives or levels improved significantly more on the game task than students using the original version of the game with all features present. Students playing game versions without changing theme, daily prizes, or end-of-session certificates and the display of lives and levels did not perform significantly differently than the comparison group. Game version did not significantly influence student motivation or performance on the object n-back task.

The effect of these game elements on training performance may seem counterintuitive at first. Why did only the “no score displayed” and “no lives or levels displayed” groups perform differently than the group with all features? And why was the removal of these motivational features associated with improved performance on the training task over the three sessions? It is worth noting that score and lives and levels were indicated on a persistent bar near the game space, a common feature in games. It is quite possible that any element that distracted the user from the challenging n-back task during the actual game would reduce performance. This is an interesting finding in light of the fact that cognitive training – and learning games in general – often include elements such as score or lives prominently in the game space. Given this possibility, one outstanding question is why the group without any motivational features did not perform significantly better than the group with all motivational features included. It is possible that although the no motivational features group did have fewer distracting elements, the exclusion of all other, non-distracting elements had a combined deleterious effect on performance. Determining whether there is a “happy medium” of motivational features that result in optimized performance is a worthwhile goal for future research. Additionally, the other motivational elements, such as awarding prizes and theme changes, did not occur during core gameplay. Over the longer term these elements may impact performance differently, but this finding provides some evidence for removing motivational elements that may be distracting from the player in the early days of a cognitive training regimen.

Overall, the lack of an effect of game variant on student self-ratings of motivation and performance on the untrained object n-back task is not surprising. Each version of the training program still appeared game-like, and the removal of any individual feature may have a minimal effect on motivation. This suggests that cognitive game designers may be able to remove some of the game elements that were found to be distracting without any negative impact on a player’s own perceptions of enjoyment and excitement. Finally, it is not necessarily surprising that only the training improvements and not performance on the object n-back task was affected by condition within the limited three-day scope of the study. It is possible that, in an experiment utilizing a much longer training experience, differences in transfer might have been observed.

Several limitations inherent to the present study should be considered. Perhaps of greatest concern is the limited sample size and significant age differences across some of the conditions. While some of the groups are adequately powered, others, due to dropout or other extenuating factors, have as few as 11 participants. Age was included as a covariate in the analyses, but the small sample sizes mean that it is difficult to fully account for the influence of age on differences in training performance. Because these findings were not corrected for multiple comparisons, and the effect sizes found were fairly small, these findings must necessarily be seen as preliminary, and, while informative of future research, not conclusive.

Additionally, this is not a true randomized controlled study – while camps were assigned to conditions randomly, all participants within each camp trained on the same variant of the game. Both of these factors are tradeoffs resulting from the real-world nature of the study; students trained amongst their peers in an actual school environment. Finally, some features of the training regimen, such as the illustrative art style and display of score at the end of each round, exist in all versions of the game. These other features may impact student performance and engagement as well, and were not examined here. The fact that some of the more subtle motivational features, such as persistent score, had a significant impact on three-day performance improvements indicates that these other features should be a focus of future research. As mentioned in the introduction, one further consideration is the possibility that certain game elements may interact with each other and that this may influence participant engagement or performance on the training task. For example, it is possible that persistent scoring is more motivating when participants receive prizes based on their score at the end of each day. This is potentially a significant issue and one that is not examined in the present study.

Besides including additional game variants, future research could also focus on the impact of these motivational features over a longer-term training regimen. It is possible that some features that impede performance on the training task in this study have less of an effect in a longer training regimen. However, given evidence that long-term improvement in the training task is necessary for transfer gains, any feature that impacts training performance is worth special consideration (Jaeggi et al., 2011). Given the fact that persistent scoring and the lives/levels feature did impact training performance, we recommend that developers of cognitive training exercise discretion when incorporating these features into their programs.

Our findings have broad implications not only for developers of cognitive training but game designers and cognitive psychologists more generally. Psychologists often make tasks game-like in an effort to drive user engagement. Likewise, within education there has recently been a movement toward game-like formative assessment to evaluate student performance (Wang, 2008). Our findings suggest that game-like elements should be added with caution. Adding game features to an already stressful testing situation may have a deleterious impact on student performance, particularly if the game features add irrelevant cognitive demands. Even seemingly innocuous features, such as displaying score or giving players a certain number of “lives,” may impact performance in a negative fashion. This does not mean that games cannot be effective teaching tools, instruments for cognitive training, or assessment mechanisms. On the contrary, this research provides further support for carefully matching game mechanics and features with the actual task. Researchers might take a look at venerable computerized training task, such as Space Fortress, and examine the impact that non-essential game-like elements included in those tasks have on performance.

While some research has supported the inclusion of game-like elements in cognitive training to improve motivation and training performance (Prins et al., 2011), our findings suggest that these features should be chosen judiciously. Combined with the results from Hawkins et al. (2013), our data suggest that game-like features may not improve the data one collects in research. Furthermore, distracting features may actually impair the participant’s ability to improve quickly at the task. Certain “motivational” elements may at best be unnecessary for driving learning on the core task, and at worst have an effect counter to what is intended by the designer. Mae West may have said “the score never interested me, only the game,” but persistent display of score, like some other motivational features, might be distracting all the same.

## Conflict of Interest Statement

The authors declare that the research was conducted in the absence of any commercial or financial relationships that could be construed as a potential conflict of interest.
